# Wedelolactone from Vietnamese *Eclipta prostrata *(L) L*. *Protected Zymosan-induced shock in Mice

**Published:** 2018

**Authors:** Trinh Tat Cuong, Giang Huy Diem, Tran Trung Doan, Nguyen Quang Huy, Nguyen Phuong, Hoang the Hung

**Affiliations:** a *Key Laboratory for Enzyme and Protein Technology, Hanoi University of Science, Hanoi, Vietnam.*; b *Faculty of Biology, Hanoi University of Science, Hanoi, Vietnam.*; c *National centre for Technological progress, Hanoi, Vietnam.*; d *Institute of scientific research in military logistics/military academy of logistic, Hanoi, Vietnam.*

**Keywords:** Wedelolactone, zymosan, sepsis, Inflammation, Reactive oxygen species

## Abstract

Wedelolactone is known to have biological activities such as anti-inflammation hepatitis, anti-hepatotoxic activity, and trypsin inhibitory effect. However, up to date, there has not been any deep study on the role of wedelolactone for zymosan-induced signaling pathways in the process of regulating the excessive inflammatory responses in host. Here, we demonstrated that wedelolactone plays an essential role for regulation of zymosan-induced inflammatory responses in murine bone marrow-derived macrophages (BMDMs). The zymosan-mediated secretion of tumor necrosis factor-α (TNF)-α), interleukin (IL)-6), and IL12p40 but not IL-10 in BMDMs was significantly inhibited by pre-treatment with wedelolactone (30 µg/mL, *P* < 0.001). Furthermore, zymosan-induced supreoxide generation, NADPH oxidase (*P* < 0.001), phosphorylation of p47phox in BMDMs were significantly reduced by pre-treatment of wedelolactone (30 µg/mL). Collectively, these data indicated that wedelolactone reduced zymosan-induced inflammatory responses. Moreover, *in-vivo* wedelolactone (30 mg/kg) was significantly rescued from zymosan-induced shock through inhibition of systemic inflammatory cytokine levels.

## Introduction

Zymosan is a rich ß-glucan component that was isolated from the cell of from Saccharomyces cerevisiae (1)*. *Zymosan is recognized by several receptors such as TLR1, TLR2, Dectin-1, the mannose receptor, and complement receptor 3 (2). This substance has been known to induce phagocytosis and inflammation both in vivo and in vitro (3). Further zymosan can induce the process of acute and chronic inflammation leading to multiple organ dysfunction syndromes (MODS) (4). Sepsis is an extreme systemic inflammatory response and is often fatal (5). Numerous attempts have been researched for the potential substances on the septic shock-induced organ damage. For example, N-acetylcystein (6) and volatile anesthetic isoflurane (ISO) (7) can suppress the interconnected ROS on the septic shock. However, therapeutic approaches are still being developed MODS model both *in-vitro* and *in-vivo*.

*Eclipta prostrata* (L) L. is a plant belonging to Asteraceae family and grows in moist places as a weed all over the world. It is widely distributed throughout India, China, Thailand, and Vietnam. Pharmacological activities of wedelolactone from *Eclipta prostrata *(L) L. has been demonstrated in vitro and *in-vivo* (8). Specially, wedelolactone is known as a wide range of bioloical activities and is used for the treatment of hepatitis and cirrhosis as viral infections (9). Recently, some researchers reported that it can inhibit growth of prostate, and pituitary cancer cells (10). Further, wedelolactone inhibits IқB kinase (IKK) complex resulting in suppression of LPS-induced caspase-11 expression (11).

However, there have not been any researches deeply done on the role of wedelolactone in zymosan-induced inflammatory responses and zymosan-induced shock. Therefore, in this study, we demonstrated the role of wedel lactone extracted from Vietnamese *Eclipta prostrata* (L) L. during the regulation of zymosan-dependent inflammatory signals in macrophages and *in-vivo *susceptibility of mice zymosan-induced shock. The results showed that wedelolactone significantly attenuated the zymosan-dependent inflammatory responses *in-vitro* and *in-vivo*. 

## Experimental

*Reagents and Abs*

Zymosan isolated from *Saccharomyces cerevisiae* (Dectin-1 agonist), was purchased from Sigma-Aldrich. Specific Abs against p38 and phospho-(Thr180/Tyr182)-p38 were purchased from Cell Signalling Technology (Beverly, MA). Anti–phospho-(Ser345)-p47phox, Ab p47phox were purchased from Santa Cruz. Biotechnology Dimethyl sulfoxide (DMSO) was purchased from Sigma. ELISA kits for IL-6, IL-10, TNF-α, and IL-12p40 were purchased from BD Pharmingen (Franklin Lakes, NJ).Also, standard wedelolactone was purchased from Sigma Aldrich. Additionally, Chloroform, methanol, toluene, acetone, formic acid, ethyl acetate, and acetonitrile were purchased from Merk.

*Cell culture*

Primary bone marrow derived-macrophages (BMDMs) were isolated from Swiss mice. The BMDMs were differentiated for 5–7 days in the medium containing Dulbecco’s modified Eagle’s medium (DMEM, Gibco-BRL, Gaithersburg, MD) with 10% L929 cell-conditioned medium (as a source of M-CSF), 10% heat- inactivated fetal

bovine serum (FBS) (Gibco-BRL), 1mM sodium pyruvate, 50 U/mL penicillin, 50 µg/mL streptomycin and 5x10^5^ M ß-mercaptoethanol, sodium pyruvate, non-essential amino acids, penicillin G (100 IU/mL), and streptomycin (100 µg/mL). 

*Plant material*

The *Eclipta prostrata* (L). L. was collected in Thai Binh provinces, Vietnam and identified (TTC01, TTC02, TTC03) by the Institute of Ecology and Biological Resources Vietnam Academy of Science and Technology (VAST), Hanoi. 

*Isolation of wedelolactone*

The leaves of *Eclipta prostrata* (L) L. were extracted with methanol by Soxhlet, as previously described (12). The solvent was removed and the residues were suspended in water separately and heated on steam bath below 80 °C. After filtrating, the aqueous phase was partitioned with ethyl acetate. The organic phase was filtered and the solvent was evaporated to reach light brown powder. The powder was subjected to fractionation by column chromatography on silica gel (glass column (1×80) cm), and the final silica length was 50 cm. The mobile phase was prepared from chloroform: methanol (70: 30). The crud and partial purified extract were subjected TLC, the solvent system (toluene: acetone: formic acid: 11: 6: 1). The purified sample and standard wedelolactone were measured by HPLC, as previously described (13). The purified sample and standard wedelolactone were diluted with methanol 10 mL. The solution was filtered through a 0.45 μm membrane filter before HPLC analysis and the injection volume was 20 µL. HPLC was performed using a Shimadzu LC-20AT pump system equipped with a Shimadzu SPD-M20A Photodiode array detector and the detection wavelength set at 351 nm. The separation was obtained using a reversed-phase column (cosmosil 5C18-AR-II 4.6 mm x 250 mm, 5μm) with the mobile phase, acetonitrile: water (35: 65 % v/v). The pH of mobile phase was adjusted to 3.2. The experiment was performed at room temperature and the flow was fixed at 1.0 mL/min.

*Experimental animals*

All experiments described in this study were performed using Swiss mice. All animal-related procedures and care were reviewed and approved by the national institute of hygiene and epidemiology (Vietnam). These mice used for the zymosan challenge were 8–10 weeks old (18-22 g). These experimental groups were matched age and sex. 

**Figure 1:**
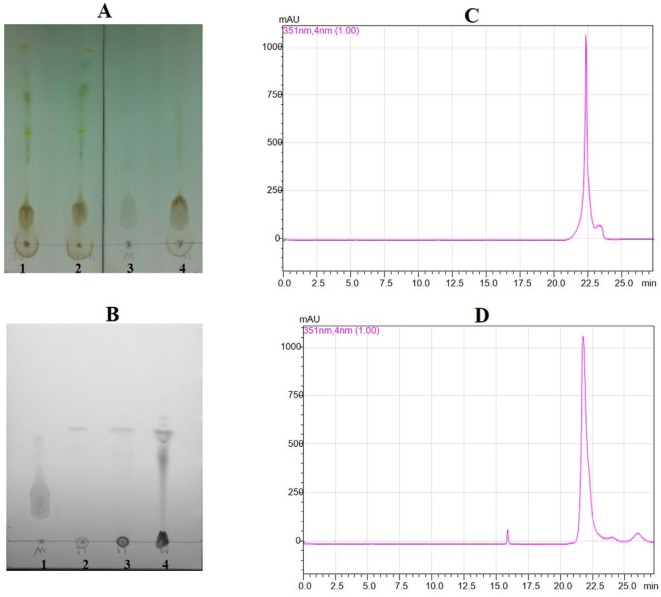
Thin layer chromatography for crude sample and partial purified sample, and HPLC for standard wedelolactone and sample wedelolactone. (A) Transfer crude sample extract in TLC (1,2,4: cude sample; 3 for standard wedelolactone). (B) Transfer partial purified sample in TLC (1: standard wedelolactone; 2, 3, 4: partial purified sample). (C) HPLC figure for standard wedelolactone. (D) HPLC Figure for sample wedelolactone

**Figure 2:**
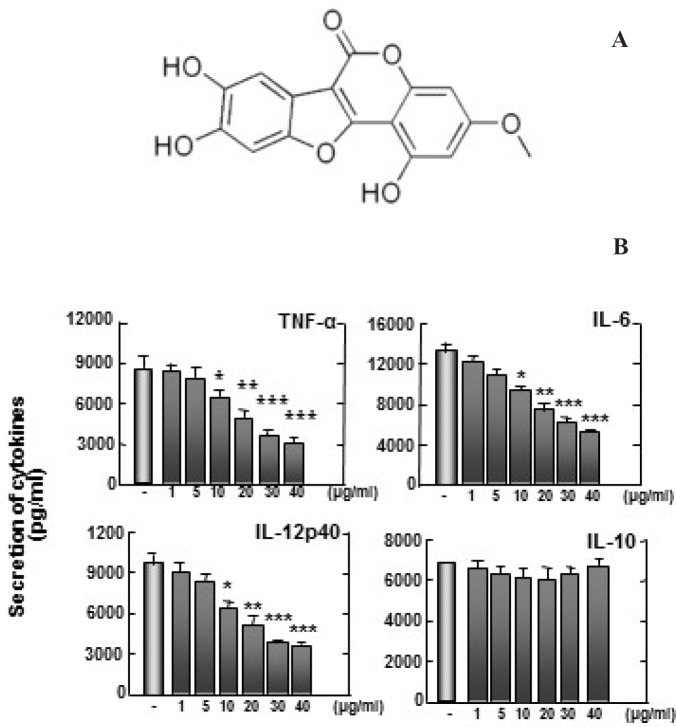
(A) Structure of wedelolactone. (B) Zymosan-induced TNF-α, IL-6, and IL-12p40 production are inhibited by wedelolactone, but not IL-10. Bone marrow-derived macrophages (BMDMs) from mice were treated with wedelolactone at concentrations of 0, 1, 5, 10, 20, 30, 40 µg/mL in DMSO 0.1% in 45 min before stimulation with zymosan. Supernatants were harvested 18 h after stimulation with 100 µg/mL zymosan to induce inflammation. Concentrations of IL-10, IL-12p40, IL-6, and TNF-α in the culture supernatants were determined by ELISA. The results are expressed the mean ± SD of five experiments. Significant differences (**P < 0.01; ***P < 0.001) compared with cultures without wedelolactone are indicated

**Figure 3:**
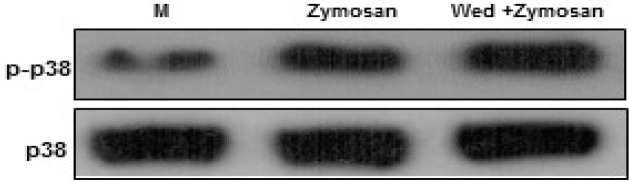
Wedelolactone negatively regulates zymosan-induced phosphorylation of p38 MAPK activation in BMDMs. The cells were pretreated solvent control (0.1% DMSO) or wedelolactone (30 µg/mL) for 45 min. Then the cells were stimulated with zymozan (100 µg/mL) for 30 min. The cells were harvested after 1 hour lysed with ice-cold lysis buffer, and subjected to western blot analysis to detect the activation of mitogen-activated protein kinases (MAPKs) (p38). M, media control; Wed, wedelolactone**. **The results are representative of three experiments

**Figure 4:**
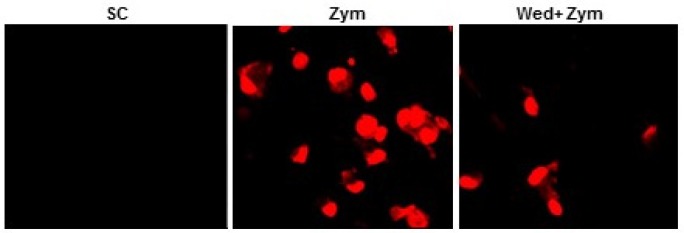
Wedelolactone inhibits the zymosan-induced production of ROS in BMDMs

**Figure 5:**
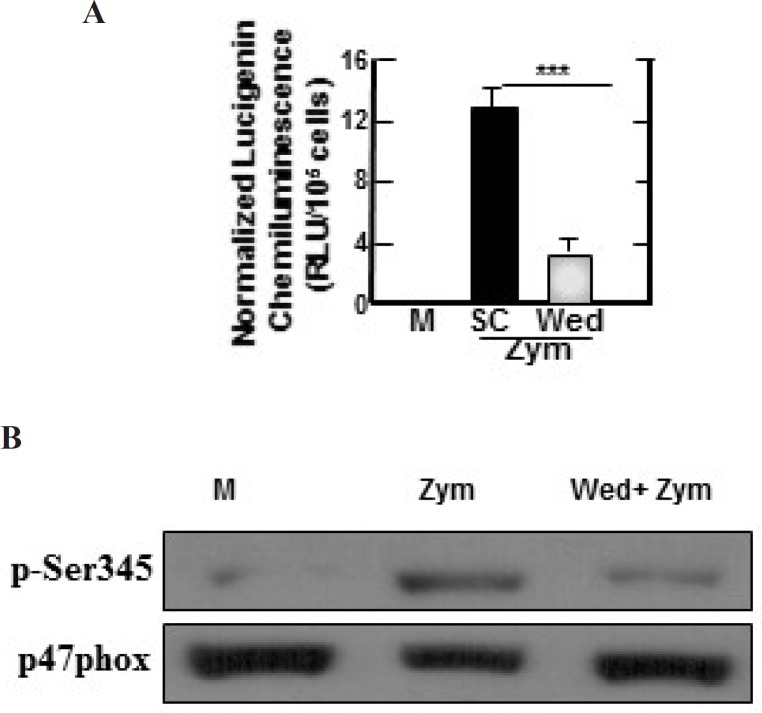
Zymosan-induced ROS-generating NADPH oxidase activities, phosphorylation of p47phox in BMDMs are inhibited by wedelolactone. (A) BMDMs were pretreated with solvent control (0.1% DMSO) or wedelolactone (30 µg/mL) for 45 min. Then the cells were stimulated with zymozan (100 µg/mL) for 60 min. NADPH oxidase activity in the cells were measured as described the materials and methods. M, media control; SC, solvent control; Wed, wedelolactone, Zym, zymosan. The results are expressed the mean ± SD of three experiments. (***, *P* < 0.001) compared with cultures without wedelolactone are indicated. (B) The cells were pretreated with solvent control or wedelolactone (30 µg/mL) for 45 min. Then the cells were stimulated with zymozan (100 µg/mL) for 15 min. The cells were lysed and analyzed by SDS-PAGE and immunoblotting with anti–phospho-Ser345–p47phox Ab (p-Ser345) or anti-p47phox Ab (p47phox). M, media control; Wed, wedelolactone; Zym, zymosan. The results are representative of three experiments

**Figure 6:**
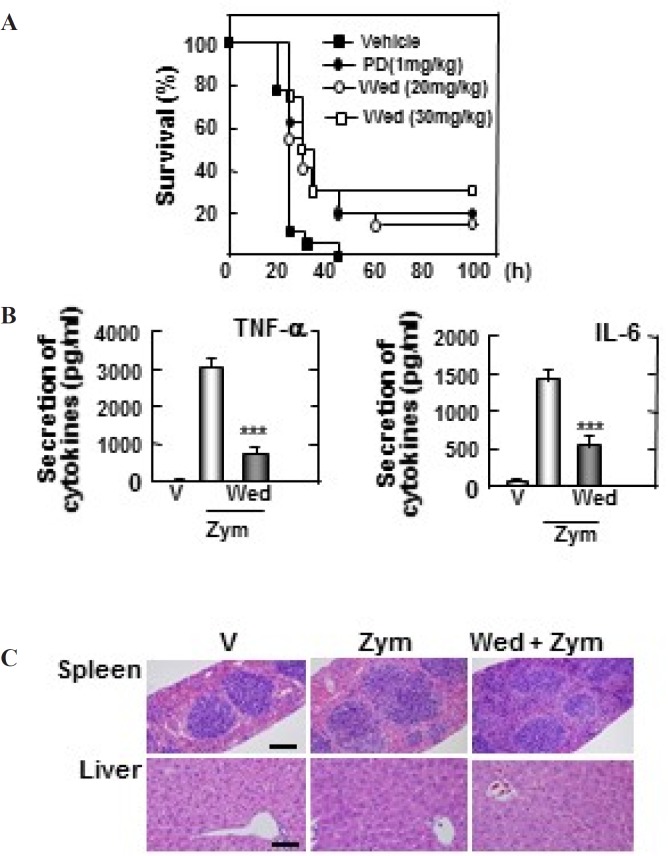
Wedelolactone protects mice against the zymosan-induced inflammatory responses. (A) Wild-type (WT) mice (n = 25 per group) were given wedelolactone (20 mg/kg) or (30 mg/kg) or vehicle or prednisone (1 mg/kg) orally for 24 h before intraperitoneal injection by zymosan (2 mg/kg). Viability was assessed every 5 h for the first 40 h and every 10 h thereafter. Wed, wedelolactone, PD, prednisone. (B) Serum levels of TNF-α, IL-6 were measured in mice that had or had not been given wedelolactone (30 mg/kg) or vehicle by using ELISA at 18 h after zymosan (2 mg/kg) injection. V, vehicle; Zym, zymosan,; Wed, wedelolactone. The data are expressed the mean ± SD of three experiments. Statistical differences (***P < 0.001) compared to the mice were not given wedelolactone are indicated. (C) Sections of spleen and liver from mice were injected with zymosan (2mg/kg) for 24 h after giving administration of wedelolactone (30 mg/kg) or vehicle. H&E staining was performed (scale bars: upper, 100 µm). V, vehicle; Zym, zymosan; Wed, wedelolactone. The images are representative of sections from five mice per group

*Enzyme-linked immunosorbent assay*

BMDMs were treated as indicated and processed for analysis by sandwich ELISA, as previously described (14). The levels of cytokines secreted by cell culture and serum were analyzed by ELISA reagent (BD Pharmingen). All assays were performed as recommended by the manufacturers.

*Western blotting*

BMDMs were treated as indicated and processed for analysis by western blotting, as previously described (15). The membranes were developed by a chemiluminescence assay (ECL; Amersham-Pharmacia).

*Measurement of intracellular ROS*

Intracellular superoxide levels were measured as previously described (15). Briefly, BMDMs were treated (60 min) with zymosan after incubating with or without wedelolactone for 45 min. Then, the cells were incubated with either 2 µM DHE (Calbiochem) for 15 min at 37°C in 5% CO_2. _The cells were examined by laser-scanning confocal microscopy (LSM 510).

*Determination of NADPH oxidase activity*

NADPH oxidase activities were evaluated by lucigenin chemiluminescence assay (5x10^-6^ mol/L lucigenin, Sigma) in the presence of its substrate NADPH (10^-4^ mol/L, Sigma) as described previously (15). 

*Experimental animals and zymosan-induced sepsis model*

Mice were injected intraperitoneal (i.p) by zymosan (2 mg/g of body weight; Sigma) was diluted in sterile phosphate-buffered saline (PBS). Wedelolactone was dissolved in a mixture of dimethyl sulfoxide (DMSO; Sigma): polyethylene glycol (PEG; Sigma) 400: distilled water (DW) (1: 4: 5). The mice were orally administered wedelolactone (20 mg/kg, 30 mg/kg) or prednisolone (1 mg/kg). The same amount of DMSO: PEG400: DW (1: 4: 5) mixture were orally administered to control group (vehicle). The mice were monitored for 5 days post-injection. The survival of mice was assessed by very 5 h for the first 40 h and every 10 h thereafter as previously described (15).

*Histological analysis*

Histological analysis was used haematoxylin and eosin as described previously (15)

*Statistical analysis*

For statistical analysis, data obtained from independent experiments are presented as mean ± SD and were analyzed by the Student’s *t*-test with a Bonferroni adjustment or ANOVA for multiple comparisons. The differences were considered significant at *P *< 0.05.

## Results and Discussion

The discovery of therapeutic or adjuvant models have ability to suppress systemic inflammation and improve survival in the patients to be important. In this study, we have shown the important regulatory role of wedelolactone in zymosan-induced inflammatory responses *in-vitro* and *in-vivo*. Wedelolactone has been reported to have various biological activities (8-11). TLC detection for the crud showed appositive result in Figure 1A. The result of the partial purified extract showed that there was one main peak (Figure 1B). The partial purified extracts were collected and concentrated for checking with standard wedelolactone by HPLC. The results indicated that there were compatibility between both sample wedelolactone and standard wedelolactone in shape, and the retention time (Figure 1C, 1D).

To examine whether wedelolactone can affect the zymosan-induced production of pro-inflammatory cytokines in BMDMs, we pretreated BMDMs with wedelolactone before stimulation with zymosan. All cytokines except IL-10 were significantly inhibited in BMDMs by pretreatment with wedelolactone (Figure 2). These findings suggest that wedelolactone regulates the zymosan-induced pro-inflammatory cytokine secretion in macrophages. Mitogen-activated protein kinases (MAPKs) activation results in induction of numerous pro-inflammatory molecules after pattern-recognition receptor signaling activation (16).

Therefore, the kinetics of p38 MAPK was analyzed in BMDMs after treatment with zymosan. The current data showed that wedelolactone negatively modulated the zymosan-induced phosphorylation of p38 MAPK (Figure 3). These data were partly correlated with previous studies (8). Wedelolactone negatively modulated activation of p38 MAPK, ERK by LPS-induced inflammation in RAW264.7 (8).

Zymosan can stimulate the production of reactive oxygen species (ROS) in myeloid cells (17) and NADPH oxidase is a main enzyme of producing superoxide in mononuclear phagocytes (18). Therefore, we investigated whether wedelolactone inhibited zymosan-induced ROS generation, and NADPH oxidase activities in macrophages. As shown in Figure 4, zymosan-induced superoxide generation was significantly inhibited in the wedelolactone-pretreated BMDM cells, and wedelolactone significantly abrogated the NADPH oxidase in cells incubated with zymosan (Figure 5A). 

Importantly, phosphorylation of p47phox is known as one of the main intracellular events associated with NADPH oxidase activation (19). As shown in Figure 5B, wedelolactone significantly blocked the phosphorylation of Ser 345 in zymosan-treated cells. Morever, zymosan induces NADPH oxidase activation by inducing the phosphorylation of p47phox in neutrophil (19).

Therefore, these data indicated wedelolactone inhibited the phosphorylation of p47phox leading the abortion of zymosan-induced NADPH oxidase in BMDMs. While ROS plays an important role in the progression of inflammatory disorders (20). Together, our data strongly suggest the potential use of wedelolactone for modulation of zymosan-induced inflammatory status through ROS-dependent pathways.

To explore the therapeutic effect of wedelolactone during zymosan-induced MODS model, the mice were pre-treated orally with or without wedelolactone (20, 30 mg/kg), prednisone (PD) (1 mg/kg), and vehicle for 24 h before intraperitoneal (i.p) injection by zymosan (2 mg/kg). Then, the survival was monitored for 5 days. As shown in Figure 6A, 100% of mice pretreated by vehicle were died at 5 days post-injection, whereas 15% of mice administered 20 mg/kg of wedelolactone (*P* < 0.001) and 30% of mice administered 30 mg/kg of wedelolactone were still alive. In addition, nearly 20% of mice that had administered PD were alive (*P* < 0.001).

The role of wedelolactone in the secretion of cytokines in mice injected with zymosan was evaluated. The contractions of TNF-α and IL-6 in the serum was significantly suppressed in mice that had administered 30 mg/kg of wedelolactone (Figure 6B). Moreover, these inflammatory changes with congestion in spleen and liver damage were significantly decreased in mice that were administered wedelolactone (Figure 6C). Together, these finding suggested that wedelolactone can suppress the inflammatory responses in zymosan-induced MODS. 

## Conclusion

This study demonstrated that wedelolactone is a potential anti-inflammatory substance isolated for the first time from medicinal plant *Eclipta prostrata* (L) L. grown in Vietnam. More importantly, the data indicated the therapeutic effects of wedelolactone in zymosan-induced inflammation in mice. Therefore, our findings may provide new opportunities for discovery of therapeutic drug to treat zymosan-induced excessive systemic inflammation.
